# Virological and clinical characteristics of hepatitis delta virus in South Asia

**DOI:** 10.1186/1743-422X-8-312

**Published:** 2011-06-20

**Authors:** Khalid Mumtaz, Umair S Ahmed, Sadik Memon, Ali Khawaja, Muhammad T Usmani, Tariq Moatter, Saeed Hamid, Wasim Jafri

**Affiliations:** 1 Section of Gastroenterology, Department of Medicine, Aga Khan University Hospital, Stadium Road, PO Box # 74800, Karachi, Pakistan; 2 Isra University Hospital, Hala Road, PO Box 313, Hyderabad, Pakistan; 3 Department of Molecular Biology, Aga Khan University Hospital, Stadium Road, PO Box # 74800, Karachi, Pakistan

## Abstract

**Background & Aims:**

There is a paucity of data on the impact of hepatitis D virus (HDV) in patients with hepatitis B virus (HBV) infection from South Asia. We studied the impact of HDV co-infection on virological and clinical characteristics.

**Methods:**

We collected data of 480 patients with HBsAg positive and a detectable HBV DNA PCR, who presented to the Aga Khan University, Karachi and Isra University in Hyderabad, Pakistan in the last 5 years. HDV co-infection was diagnosed on the basis of anti-HDV. ALT, HBeAg, HBeAb and HBV DNA PCR quantitative levels were checked in all patients. We divided all patients into two groups based on anti-HDV, and compared their biochemical, serological & virological labs and clinical spectrum. Clinical spectrum of disease included asymptomatic carrier (AC), chronic active hepatitis (CAH), immuno-tolerant phase (IP), and compensated cirrhosis (CC).

**Results:**

HDV co-infection was found in 169 (35.2%). There were 164 (34.6%) HBeAg positive and 316 (65.4%) HBeAg negative patients. Mean ALT level was 66 ± 73 IU. 233 (48.5%) had raised ALT. HBV DNA level was ≥ 10e5 in 103(21.5%) patients. Overall, among HBV/HDV co-infection, 146/169 (86.4%) had suppressed HBV DNA PCR as compared to 231/311 (74.3%) patients with HBV mono-infection; p-value = 0.002. Among HBeAg negative patients 71/128(55.5%) had raised ALT levels among HBV/HDV co-infection as compared to 71/188 (37.8%) with HBV mono-infection (p-value = 0.002); levels of HBV DNA were equal in two groups; there were 27/128 (21%) patients with CC among HBV/HDV co-infection as compared to 23 (12%) in HBV mono-infection (p-value = 0.009); there were less AC (p-value = 0.009) and more CAH (p-value = 0.009) among HBV/HDV co-infection patients. Among HBeAg positive patients, serum ALT, HBV DNA levels and the spectrum of HBV were similar in the two groups.

**Conclusions:**

HBV/HDV co-infection results in the suppression of HBV DNA. A fair proportion of HBV/HDV co-infected patients with HBeAg negative have active hepatitis B infection and cirrhosis as compared to those with mono-infection.

## Introduction

Hepatitis delta virus (HDV) is a satellite RNA virus that depends on the envelope protein of the hepatitis B virus (HBV) to enter the hepatocytes and assemble new HDV particles [1]. Worldwide, more than 350 million people are considered to have chronic HBV infection, and 15-20 million of these individuals are thought to be co-infected or super-infected with HDV [2].

Hepatitis delta is considered to be the most severe form of viral hepatitis, often leading to the rapid development of liver cirrhosis. Furthermore HDV infection has also been linked with a higher risk for the development of hepatocellular carcinoma [3].

The infection was endemic in the 1970s throughout Southern Europe, and was responsible for a substantial proportion of cases of HBsAg- positive liver disorders [4,5]. However, the prevalence of HDV had substantially declined in Italy from 23% in 1987 to 8.3% in 1997 as reported by Stroffolini et al [6]. A similar decline was noted in Taiwan, with prevalence decreasing from 23.7% in 1983 to 4.2% in 1996 [7], as well as in Spain and Turkey [8]. This decline in prevalence of HDV infection was achieved by enhancing awareness among the general public and by measures taken for vaccination against hepatitis B in these countries. In Pakistan, however, viral hepatitis remains a serious health problem. We have reported the country-wide prevalence of HDV infection in hepatitis B surface antigen (HBsAg)-positive individuals to be 16.6% [9]. Very recently, a comprehensive report on the epidemiology of hepatitis delta in the Asia-Pacific region was published by Abbas et al [10]. According to this report, prevalence from different parts of Asia is variable, and ranges from 3-10% in India, 2-20% in Iran, 18% in Afghanistan and 3-8% in Saudi Arabia.

There are seven HDV genotypes described, and their nomenclature is defined as type I-VII; various genotypes are reported to be associated with different long term outcomes of infection [11]. Recently Moatter et al reported genotype 1 of HDV and genotype D of HBV from Pakistan [12], which has also been confirmed on a larger scale by our group (13).

HBV infection is associated with a broad spectrum of clinical manifestations, ranging from an asymptomatic carrier (AC) state to acute liver failure. It can also manifest in diverse forms of chronic infection, including the immune tolerant phase (IP), chronic active hepatitis B (CAH), compensated liver cirrhosis (CC), and hepatocellular carcinoma (HCC).

Co-infections of hepatitis B with multiple hepatitis viruses are associated with diverse patterns of reciprocal inhibition of viral replication. Delta hepatitis occurs due to co-infection of HBsAg positive patients with hepatitis delta virus. There are inconsistent reports on the role of each virus in the pathogenicity of HBV/HDV infection. Some reports suggest that the activity of liver disease is mainly due to HDV [13-16] while others implicate hepatitis B virus, regardless of the levels of HBV DNA, in the aggressive nature and progression of disease [17]. In studies from Europe, HDV has frequently been shown to suppress HBV replication [18,19], and 70-90% of patients with hepatitis D are hepatitis B e antigen (HBeAg) negative, with low serum levels of HBV DNA. However, despite this influence of HDV on HBV, 15-30% of patients with hepatitis D are HBeAg and/or HBV DNA positive.

There is no data, to our knowledge, on the characteristics and impact of hepatitis delta virus on hepatitis B virus infection and its spectrum of diseases from South Asia. The aim of this study was to investigate the virological and clinical characteristics of patients infected with HBV/HDV infection in two large tertiary care centers of Pakistan.

## Methods

### Patients' characteristics

We conducted this study prospectively in patients seen at the Aga Khan University Hospital (AKUH), Karachi and Isra University Hospital, Hyderabad, Pakistan. Both hospitals are situated in the province of Sindh and serve as main tertiary care centers located in the southern part of Pakistan, which is among the largest countries of South Asia; it represents 30% of the population of this region. Each year approximately half a million patients visit the out-patient clinics and 45,000 are managed as in-patients in the different wards of these two hospitals. The AKUH laboratory has 189 collection centers all around the country including 07 in Hyderabad where the samples are collected and transported to the central laboratory in Karachi for processing.

We identified 2455 HBsAg positive patients and checked their HBV DNA PCR by qualitative methods from 2005 to 2009 at these two centers. Out of 2455 patients with a reactive HBsAg, 480 patients with a positive HBV DNA PCR underwent testing for anti-HDV and quantitative HBV DNA PCR assay. HDV infection was defined by positive anti-HDV results. Demographics and physical findings were recorded in all patients with or without HDV co-infection prior to the start of any anti-viral treatment.

### Biochemical and virological markers

Serum samples of all patients were tested for biochemical parameters including complete blood count, total bilirubin, ALT, alkaline phosphatase, creatinine and prothrombin time (PT), by standard laboratory methods. Upper limit of ALT was 55 IU/L for men and 33 IU/L for women. Serological tests conducted were qualitative HBsAg, HB core IgG, HBeAg, anti-HBe, anti-HDV by EIA assay (Chicago, IL, USA) and anti-HCV by ELISA-3 in all included patients during their visits to clinic within six months. All the biochemical, serologic and virological tests from the two centers was processed in the central clinical laboratory at the Aga Khan University Hospital, Karachi.

HBV DNA PCR qualitative analysis was performed by Cobas Amplicor HBV Monitor (Roche Diagnostic Systems, Basel, Switzerland) with a lower detection limit of 500 copies/ml. We considered the value of the qualitative PCR detectable to be ≤10 ^2^. All these patients were subsequently tested for quantitative HBV DNA PCR assay (RoboGene HBV DNA Quantification Kit, TripleHyb version, Leipzig, Delitzscher Str, Germany) and anti-HDV testing. Quantitative HBV DNA PCR has the lower limit of detection of approximately 1000 (> 10 ^2^) copies/ml. HDV RNA PCR qualitative assay (Roche Diagnostics, USA) could only be checked in 49 patients with HDV co-infection as this test was not available in early part of the study in our laboratory.

HDV RNA was isolated from patients' serum samples by High Pure Viral RNA isolation kit, according to the manufacturer's instructions (Roche Diagnostics, USA). RNA was eluted from spin columns provided with the kit in sterile nuclease free water and stored at -80 °C until further analysis. Later, RNA samples were reverse transcribed into cDNA using 1st Strand cDNA Synthesis Kit for RT-PCR (Roche Diagnostics, USA). Briefly, cDNA mix consisted of reaction buffer containing 5 mM MgCl_2_, R NA, random primers, 50 units RNAse inhibitor and AMV reverse transcriptase. The reaction was carried out for 90 min at 42 °C in a thermal cycler. The resulting cDNA was amplified with sequence specific primers for HDV. The amplified products were separated on a 1.5% agarose gel and a 400 bp product indicated the presence of HDV in the sample. To monitor the quality of the assay in each test run, both negative and positive controls were included [20].

Abdominal ultrasound of all included patients was performed for the assessment of echo-texture, size and margins of liver & spleen and for features suggestive of portal hypertension. Stages of liver disease such as those with or without cirrhosis were identified on clinical and/or biochemical laboratory parameters along with ultrasonological findings.

### Spectrum of hepatitis virus infection

480 hepatitis B patients with or without HDV infection were categorized into four clinical stages: **(1) Asymptomatic carrier (AC) **- patients were clinically asymptomatic with hepatitis B e antigen (HBeAg) negative, serum HBV DNA levels are < 10^5 ^and normal serum ALT levels (at least 2 ALT values in the previous 6 months follow-up). **(2) Immune-tolerant phase (IP) **- patients were asymptomatic with hepatitis B e antigen (HBeAg) positive or negative, high serum HBV DNA levels (≥ 10^5^) and normal serum ALT levels (at least 2 ALT values in the previous 6 months). **(3) Chronic active hepatitis B (CAH) **- patients may be symptomatic or asymptomatic with hepatitis B e antigen (HBeAg) positive or negative, with raised serum HBV DNA PCR and ALT levels (at least 2 ALT values above ULN in the previous 6 months). These patients have no evidence of cirrhosis on clinical, biochemical laboratory parameters along with ultrasonological examination. **(4) Compensated liver cirrhosis (CLC) - **patients may be symptomatic or asymptomatic with hepatitis B e antigen (HBeAg) positive or negative, whatever are their serum HBV DNA levels, and ALT levels in the previous 6 months. These patients have evidence of cirrhosis identified on clinical, and/or biochemical laboratory parameters along with abdominal ultrasound examination [21,22]. HDV co-infection-patients with anti-HDV reactive and/or HDV RNA PCR detectable [23].

### Exclusion criteria

We excluded patients (1) under the age of 14 years, (2) with decompensated liver disease (defined by a serum bilirubin more than 2.5 times the upper limit of normal; prothrombin time > 3 seconds compared with control; serum albumin < 2.5 g/dl; or a history of ascites, variceal hemorrhage or hepatic encephalopathy or hepatocellular carcinoma (3) with evidence of liver disease due to another etiology such as hepatitis C, metabolic liver disease or autoimmune liver disease, (4) history of use of hepatotoxic drugs and (5) patients with multiple infections (i.e. HBV, HDV, HIV and HCV).

This study was approved by the Ethics Review Committee (ERC) of Aga Khan University Hospital.

### Statistical Analysis

Statistical analysis was performed using the Statistical Package for Social Science SPSS (Release 17.0, standard version, copyright ^© ^SPSS; 1989-02). Descriptive analysis was performed for demographic and clinical features; results were presented as mean ± standard deviation for quantitative variables and number (percentages) for qualitative variables.

A descriptive analysis was performed for all patients with HBsAg positive along with HBV DNA PCR detectable. To determine any statistical difference, data were divided into HBeAg positive and HBeAg negative sub-groups. The two groups were compared for demographic and laboratory parameters. In HBeAg positive patients' comparison was conducted between HBV/HDV co-infection and HBV mono-infection patients; similar comparison was performed for the HBeAg negative patients. Chi-square test and Fisher's exact test were used for categorical variables, while the independent sample test was used for numerical variables. All p-values were two sided and considered as statistically significant if < 0.05.

## Results

A total of 2455 HBsAg positive patients were checked for HBV DNA PCR by qualitative assay; 480 (19.5%) patients with HBV DNA PCR by qualitative assay were eligible for inclusion in this study. HBV DNA PCR quantitative assays and anti-HDV serology was checked in all these patients.

### Virology and HBV DNA PCR assays

Overall, there were 398 (82.9%) males with a mean age of 33 ± 12.5 years. Mean HBV DNA PCR levels was 1.9 × 10 ^7 ^± 1.2 × 10 ^8 ^copies/ml. There were 169/480 (35.2%) patients with HBV/HDV co-infection.

Overall, the HBV DNA PCR assay above 1000 (> 10^2^) copies/ml was reported in 294 (61%) while rest have ≤10^2 ^copies/ml. Moreover, higher HBV DNA levels of ≥ 10^5 ^copies/ml were found in 103 (21.5%), while < 10^5 ^in 377 (78.5%) patients; table 1.

**Table 1:** Characteristics of study population in HBeAg positive and negative patients

	Total n = 480	HBeAg positive n = 164	HBeAg negative n = 316	p value
Age, years	33 ± 12.53	30 ± 13.36	34.28 ± 11.87	< 0.001

				

Gender				

Male	398(82.9)	138(84.1)	260(82.3)	0.60

Female	82(17.1)	26(15.9)	56(17.7)	

				

ALT	66.07 ± 72.83	73.95 ± 79.04	61.98 ± 69.16	0.10

Median ALT	38(9-500)	42(9-500)	35(10-496)	

Normal	247 (51.5)	73(44.5)	174(55.1)	0.02

Raised	233 (48.5)	91(55.5)	142(44.9)	

				

HBV DNA level				

≥ 10e^5^	103 (21.5)	83 (50.9)	20 (15.3)	< 0.001

< 10e^5^	377 (78.5)	80 (49.1)	111 (84.7)	

				

Anti HDV				

Reactive	169 (35.2)	41 (25)	128 (40.5)	0.001

Non reactive	311 (64.8)	123 (75)	188 (59.5)	

				

HDV RNA qualitative				

Positive	49 (10.2)	7 (5.7)	42 (13.8)	< 0.001

Negative	67 (14)	7 (5.7)	60 (19.7)	

				

HBV stage				

Carrier of HBV	143 (29.8)	0	143 (45)	< 0.001

Chronic active HBV	185 (38.5)	75 (45.7)	110 (34.8)	

Compensate cirrhosis with HBV	72(15)	22 (13.4)	50 (15.8)	0.48

Immunotolerant phase	80 (16.7)	60 (36.6)	20 (6.3)	NS

				

NS = not significant

### HBeAg positive versus HBeAg negative patients

Out of 480 patients, 164 (34.6%) were HBeAg positive and 316 (65.4%) HBeAg negative. We found that HBeAg positive patients were younger (30 ± 13.3 years) as compared to HBeAg negative (34.3 ± 11.9 years); p-value = < 0.001. ALT levels above upper limit of normal (ULN) were found in 91 (55.5%) HBeAg positive as compared to 142 (45%) among HBeAg negative group; (p-value = 0.02). Moreover, the level of HBV DNA PCR was ≥ 10^5 ^copies/ml in 83 (51%) patients with HBeAg positive as compared to 20 (15.3%) among HBeAg negative, (p-value < 0.001); table 1.

HBeAg positive patients have raised ALT and higher HBV DNA levels as compared to HBeAg negative.

### HDV infection and ALT levels

Mean ALT levels in our study population were 66 ± 73 IU. Moreover, mean ALT in patients with HBV/HDV co-infection was 74.3 ± 76.7 IU as compared to 61.6 ± 70.3 IU in HBV mono-infection; (p-value = 0.06). Furthermore, there were 96/169 (56.8%) patients with raised ALT in HBV/HDV co-infection group as compared to 137/311 (44%) in HBV mono-infection, (p-value 0.008); table 2. Overall HBV/HDV co-infection patients have raised ALT levels.

**Table 2:** Clinical and laboratory parameters in HDV positive and negative patients

	HDV positive n = 169	HDV negative n = 311	p value
Age (years)	32.74	33	0.88

Sex-male	136 (80.5%)	262 (84.2%)	0.29

ALT-mean	74.3 ± 76.7	61.6 ± 70.3	0.06

ALT normal	73 (43.2%)	174 (55.9%)	0.008

ALT raised	96 (56.8%)	137 (44.1%)	

HBeAg positive	41 (24.3%)	123 (39.5%)	0.001

HBeAg negative	128 (75.7%)	188 (60.5%)	

Mean HBV DNA levels			

HBV DNA > 10^5^	23 (13.6%)	80 (25.7%)	0.002

HBV DNA < 10^5^	146 (86.4%)	231 (74.3%)	

**HBV stages**			

Carrier	45 (26.6%)	98 (31.5%)	0.003

Chronic active HBV	73 (43.2%)	112 (36%)	

Compensate cirrhosis	34 (20.1%)	38 (12.2%)	0.02

Immunotolerant phase	17 (10.1%)	63 (20.3%)	NS

NS = not significant

Among HBeAg positive patients mean ALT was 74 ± 79 IU; 91/164 (55.5%) patients had a raised ALT above the ULN. Out of 164 patients with positive HBeAg, 41 patients had a HBV/HDV co-infection, while 123 had HBV mono-infection. Of the patients with HDV co-infection (n = 41), 25/41 (61%) had raised ALT, while in patients with HBV mono-infection (n = 123), 66 (53.7%) patients had raised ALT level; (p value = 0.41); table 3. Among patients with raised ALT, 47 (51.6%) had HBV DNA level ≥ 10^5 ^while 44 (48.4%) had a HBV DNA level < 10^5^; (p-value = 0.76).

**Table 3:** Descriptive characteristics of HBeAg positive patients (n = 164) in patients with HBV/HDV co-infection and HBV mono-infection

	HDV Positive n = 41	HDV Negative n = 123	p value
Age, years	29 ± 8.85	30.34 ± 14.53	0.44

Gender-male	34 (82.9%)	104 (84.6%)	0.80

Male	34 (82.9)	104 (84.6)	0.80

Raised ALT	25 (61)	66 (53.7)	0.41

Mean ALT	72.2 ± 70.7	74.5 ± 82	0.87

**HBV DNA level**			

≥ 10e^5^	17 (41.5)	66 (54.1)	0.17

< 10e^5^	24 (58.5)	57 (46.3)	

**Spectrum of HBV:**			

Carrier of HBV	00	7 (5.7)	0.54

Chronic active HBV	21 (51.2)	54 (43.9)	NS

Compensate cirrhosis with HBV	7 (17.1)	15 (12.2)	NS

Immunotolerant phase	12 (29.3)	48 (39)	NS

NS = not significant

Among HBeAg positive patients, HBV/HDV co-infection has no affect on ALT and HBV DNA levels.

Among HBeAg negative patients, mean ALT was 62 ± 69 IU; 142/316 (45%) had raised ALT level.

In this group mean ALT was 75 ± 79 in HBV/HDV co-infection as compared to 53 ± 60.3 among HBV mono-infection patients, (p-value = 0.009). Moreover, ALT levels were above ULN in 71/128 (55.5%) with HBV/HDV co-infection as compared to 71/188 (37.8%) among HBV mono-infection; (p-value = 0.002); table 4. Among patients with raised ALT, 12 (8.5%) had a HBV DNA level ≥ 10^5 ^while 130 (91.5%) had a HBV DNA level < 10^5 ^copies/ml (p-value = 0.16).

**Table 4:** Descriptive characteristics of HBeAg negative patients (n = 316) in patients with HBV/HDV co-infection and HBV mono-infection

	HDV Positive n = 128	HDV Negative n = 188	p value
Age, years	34 ± 11.3	34.5 ± 12.2	0.63

Gender-Male	102(79.7)	158(84)	0.32

Raised ALT	71(55.5)	71(37.8)	0.002

			

HBV DNA Positive	41(32.3)	74(39.4)	0.20

			

HBV DNA level			

≥ 10e^5^	6(11.5)	14(17.7)	0.32

< 10e^5^	122(95.3)	174(92.6)	

			

Cirrhosis	27(21.1)	23(12.2)	0.03

			

HBV stage			

Carrier of HBV	44(34.4)	92(48.9)	0.009

Chronic active HBV	52(40.6)	58(30.9)	

Compensate cirrhosis with HBV	27(21.1)	23(12.2)	NS

Immunotolerant phase	5(3.9)	15(8)	NS

NS = not significant

Among HBeAg negative patients, HBV/HDV co-infection is associated with raised ALT levels, but ALT levels were not directly proportionate to HBV DNA levels.

### HDV infection and HBeAg status

A large proportion of patients with HBV/HDV co-infection had HBeAg negative (128/169; 75.7%) disease, as compared to HBV mono-infection (188/311; 60.5%); p-value 0.001.

Among HBeAg positive patients, 41/169 (24.3%) had HBV/HDV co-infection. The distribution of age, gender, ALT and HBV DNA PCR levels were similar among the two groups. Furthermore, the spectrum of hepatitis B disease was also similar in both groups; table 3.

**Figure 1:**
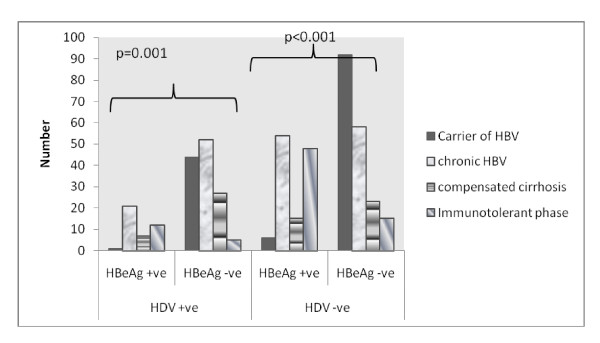
**Comparison of spectrum of hepatitis B related liver diseases in patients with HBV/HDV co-infection and HBV mono-infection, based on HBeAg status**.

HBV/HDV co-infection has no implications on the HBV DNA PCR as well as the spectrum of liver disease. Table 3; Figure 1.

Among HBeAg negative patients, 128/316 (40.5%) had HBV/HDV co-infection. HBV DNA PCR levels were equally distributed in the two groups of patients (table 2). Patients with HBV/HDV co-infection had more severe liver disease - compensated liver cirrhosis was present in 27 (21%) as compared to 23 (12%) among HBV mono-infection; p-value = 0.03. Similarly, chronic active hepatitis (CAH) in 52 (40.6%) patients with HBV/HDV co-infection as compared to 58 (31%) without it; (p-value = 0.009); table 4; Figure 1

Among HBeAg negative patients with HBV/HDV co-infection, severe form of liver disease is seen, though HBV DNA levels were equally distributed, suggesting that HDV is actively involved in the progression of liver disease.

### HDV infection and HBV DNA quantitative assays

**Figure 2:**
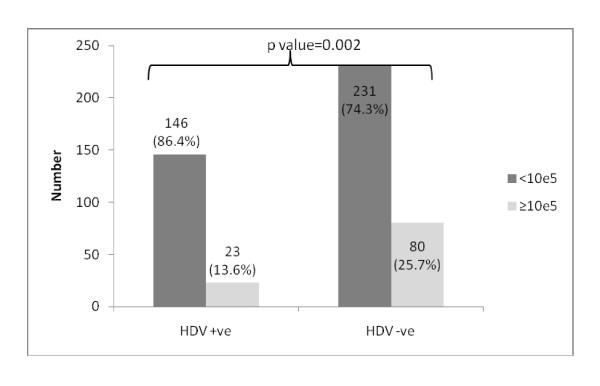
**Showing significant HBV DNA levels suppression in patients with HBV/HDV co-infection**.

**Figure 3:**
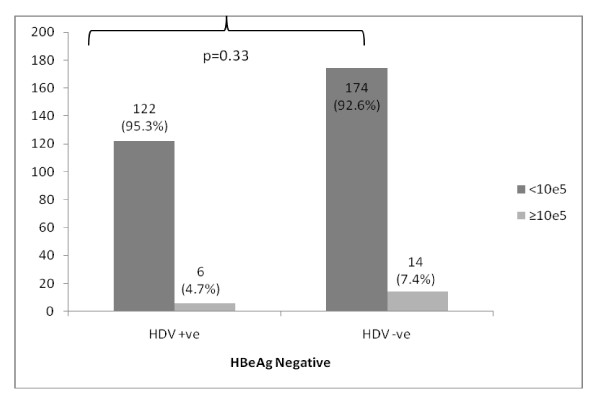
Showing absence of suppression of HBV DNA PCR levels in patients with HBV/HDV co-infection with HBeAg negative disease

Overall levels of HBV DNA PCR < 10^5 ^was found in 146 (86.4%) HBV/HDV co-infected patients as compared to 231 (74.3%) HBV mono-infection (p-value = 0.002); table 1b. However, this suppression of HBV DNA levels in HBV/HDV co-infection patients was independent of the HBeAg status; table 3 &4 and Figure 2 &3.

This suggests presence of HBV/HDV co-infection suppresses HBV DNA levels.

### HDV infection and clinical spectrum

The details of demographics, baseline laboratory parameters and distribution of spectrum of hepatitis B related liver disease is shown in table 1. There were 73/169 (43%) patients with chronic active hepatitis B (CAH) among HBV/HDV co-infection as compared to 112/311 (36%) among HBV mono-infection; (p-value = 0.003). Similarly there were 34 (20%) patients with compensated liver cirrhosis (CLC) among HBV/HDV co-infection as compared to 38 (12%) in HBV mono-infection group; p-value = 0.02. We found most asymptomatic carrier and immune-tolerant hepatitis B patients in HBV mono-infection group (table 1). HBV/HDV co-infection produces more severe clinical spectrum of liver disease. Moreover, this impact of HBV/HBV co-infection is more marked in HBeAg negative patients as already mentioned above.

## Discussion

This is the largest report that we know on viral characteristics of hepatitis delta virus infection, recruited at two centers of Pakistan which represent a robust population of South Asia. In particular, data of HBeAg positive patients is limited globally. HBV patients can be simultaneously infected by delta virus, as the source of transmission of both viruses is same and results in a severe form of liver disease. There are variable reports, as to which virus is actively responsible for liver disease in patients with chronic HDV infection - HBV or HDV? [14-17]. We designed this study to examine the effects of HBV/HDV co-infection on ALT, HBeAg status and HBV DNA PCR levels, in addition to the different spectrums of hepatitis B related liver disease. Our present study showed that 35.2% of patients had HBV/HDV co-infection. We published an epidemiological survey of HDV prevalence in 2005. This survey included 8721 HBV patients over 14 years of age and tested for anti-HDV antibody from all over the country. The HDV prevalence was found to be 16.6% [9]. Higher frequency of HDV co-infection in this report could have been due to a selection bias as reported by Seetlani et al [24].

In our present study, majority of patients with HBV/HDV co-infection were young males, which is similar to earlier studies from Italy [25], and Pakistan [9]. One possible explanation could be the higher rate of intravenous drug abuse in this cohort of patients in the developed countries, and therapeutic injections with contaminated needles and vertical transmission in the developing world [26]. The mean age of cirrhotic HDV patients was also younger than that of cirrhotic HBV patients without HDV. These findings indicate the greater severity of chronic HDV infection than chronic HBV infection alone.

Our study shows that patients with HBV/HDV co-infection have raised ALT levels and suppressed HBV DNA levels as compared to HBV mono-infection. Moreover, HBV patients with HBeAg positive disease regardless of their HDV status have raised ALT levels and high HBV DNA levels as compared to HBeAg negative patients. Interestingly, we observed that in HBeAg positive patients, ALT and HBV DNA levels were equally distributed among HBV/HDV co-infection and HBV mono-infection patients. It seems that among HBeAg positive patients HBV/HDV co-infection has no impact on the HBV DNA levels.

Contrary to HBeAg positive group, among HBeAg negative patients HBV/HDV co-infection is associated with raised ALT levels, but ALT levels were not correlated to high HBV DNA levels. Among HBeAg negative patients with HBV/HDV co-infection, a more severe spectrum of liver disease was seen, although the HBV DNA level was low. This would suggest that HDV is actively involved in progress of liver disease.

Our study also shows that HBV/HDV co-infection, suppresses hepatitis B virus. HBV/HDV co-infection produces a more aggressive disease as compared to mono-infection with hepatitis B. This has also been reported in many studies [14-16,23] for HBeAg negative patients.

Furthermore, HBeAg, a marker of viral replication, was found in a higher percentage of patients with HBV mono-infection as compared to HBV/HDV co-infection, which was also noted in our study. It has also been well documented that the difference in HBV genotype affects the prevalence of HBeAg and the replication level of HBV [27,28]. HBV genotype B is reported to be associated with HBeAg negativity and a lower viremia level. Furthermore, certain HDV strains, like HDV genotype II are associated with more mild liver disease. Correlation between HDV genotypes and HBV replication however remains largely unknown.

The main mechanism of inhibition of viral replication in HBV infection is not cytotoxicity against infected hepatocytes but rather the secretion of inhibitory cytokines like interferon-gamma, interferon-alpha or TNF-alpha. HDV proteins inhibit HBV replication by trans-repressing its enhancers and by trans-activating the IFN-alpha-inducible MxA gene [29].

Individuals having HBV/ HDV co-infection may have a more severe acute disease and higher risk of fulminant hepatitis [14-16]. It is observed that most of the individuals infected with HDV develop the chronic form of the disease, and in approximately 80% of these individuals the chronic HDV infection progresses to cirrhosis within 5-10 years [30]. This observation is also noted in our HBV/HDV co-infection patients. HBV/HDV co-infection showed a several fold increase in progression of chronic hepatitis to cirrhosis (p-value 0.003) as compared to HBV mono-infection.

In conclusion, we found that HBV/HDV co-infection results in suppression of hepatitis B virus. Patients with HBV/HDV co-infection with negative HBeAg status demonstrate a severe spectrum of liver disease. Due to limited and ineffective treatment options for the treatment of HBV/HDV infection our main focus must be on the prevention of the disease. While the prevalence of HDV is showing a decreasing trend globally, it is important to realize that globalization and increasing migratory fluxes are major challenges and HDV will not be controlled adequately unless HBV is brought under control in areas with high prevalence.

## Competing interests

The authors declare that they have no competing interests.

## Authors' contributions

KM contributed in terms of original idea, study design, writing protocol, organizing logistics, and editing the article. USA and SM contributed to study design, logistics, caring for patients under study and editing the article. AK and MTU contributed by collecting data, literature search, writing of the article with first two authors. TM, SH and WJ contributed in study design, care of study patients, and editing of the article. All authors read and approved the final manuscript.

## References

[B1] Taylor JM Hepatitis delta virus Virology 2006 344 71 76 10.1016/j.virol.2005.09.033 16364738

[B2] Hadziyannis SJ Review: hepatitis delta J: Gastroenterol Hepatol 1997 12 289 298 10.1111/j.1440-1746.1997.tb00424.x 9195369

[B3] Caturelli E Castellano L Fusilli S Coarse nodular US pattern in hepatic cirrhosis: risk for hepatocellular carcinoma Radiology 2003 226 691 697 10.1148/radiol.2263011737 12601208

[B4] Rizzetto M Purcell RH Gerin JL Epidemiology of HBV associated delta agent: geographical distribution of antidelta and prevalence in poly-transfused HBsAg carriers Lancet 1980 1 1215 18 6104036 10.1016/s0140-6736(80)91678-5

[B5] Cotrina M Buti M Jardi R Hepatitis delta genotypes in chronic delta infection in the northeast of Spain (Catalonia) J Hepatol 1998 28 971 7 9672172 10.1016/s0168-8278(98)80345-0

[B6] Stroffolini T Ferrigno L Chialdea L Incidence and risk factors of acute delta hepatitis in Italy: results from a national surveillance system J Hepatol 1994 21 1123 6 10.1016/S0168-8278(05)80629-4 7699238

[B7] Gaeta GB Stroffolini T Chiaramonte M Chronic hepatitis D: a vanishing disease? An Italian multicenter study Hepatology 2000 32 824 7 10.1053/jhep.2000.17711 11003629

[B8] Degertekin H Yalcin K Yakut M The prevalence of hepatitis delta virus infection in acute and chronic liver diseases in Turkey: an analysis of clinical studies Turk J Gastroenterol 2006 17 25 34 16830274

[B9] Mumtaz K Hamid SS Adil S Epidemiology and clinical pattern of hepatitis delta virus infection in Pakistan J Gastroenterol Hepatol 2005 20 1503 1507 10.1111/j.1440-1746.2005.03857.x 16174065

[B10] Abbas Z Jafri W Raza S Hepatitis D: Scenario in the Asia-Pacific region World J Gastroenterol 2010 16 5 554 562 10.3748/wjg.v16.i5.554 20128022 PMC2816266

[B11] CW Su Huang YH Huo TI Genotypes and viremia of hepatitis B and D viruses are associated with outcomes of chronic hepatitis D patients Gastroenterology 2006 130 1625 1635 10.1053/j.gastro.2006.01.035 16697726

[B12] Moatter T Abbas Z Shabir S Clinical presentation and genotype of hepatitis delta in Karachi World J Gastroenterol 2007 13 2604 2607 17552010 10.3748/wjg.v13.i18.2604 PMC4146823

[B13] Mumtaz K Hamid S Ahmed S A study of genotypes, mutants and nucleotide sequence of hepatitis B virus in Pakistan Hepatitis Monthly 2011 11 1 25 29 PMC3206661 22087110

[B14] Sakugawa H Nakasone H Nakayoshi T Hepatitis B virus concentrations in serum determined by sensitive quantitative assays in patients with established chronic hepatitis delta virus infection J Med Virol 2001 65 478 484 10.1002/jmv.2061 11596082

[B15] Yamashiro T Nagayama K Enomoto N Quantification of the level of hepatitis delta virus RNA in serum, by real-time polymerase chain reaction - and its possible correlation with the clinical stage of liver disease J Infect Dis 2004 189 1151 1157 10.1086/382133 15031781

[B16] Hadziyannis SJ Sherman M Liberman HM Liver disease activity and hepatitis B virus replication in chronic hepatitis delta antigen-positive hepatitis B virus carriers Hepatology 1985 5 544 547 10.1002/hep.1840050404 4018727

[B17] Su CW Huang YH Huo TI Genotypes and viremia of hepatitis B and D viruses are associated with outcomes of chronic hepatitis D patients Gastroenterology 2006 130 1625 1635 10.1053/j.gastro.2006.01.035 16697726

[B18] Rodriguez F Buti M Role of hepatitis B, C, and D viruses in dual and triple infection: influence of viral genotypes and hepatitis B precore and basal core promoter mutations on viral replicative interference Hepatology 2001 34 404 410 10.1053/jhep.2001.26511 11481626

[B19] Coppola N Scolastico C Virologic and clinical expressions of reciprocal inhibitory effect of hepatitis B, C, and delta viruses in patients with chronic hepatitis Hepatology 2000 32 1106 1110 10.1053/jhep.2000.19288 11050062

[B20] Flodgren E Bengtsson S Knutsson M Recent high incidence of fulminant hepatitis in Samara, Russia: molecular analysis of prevailing hepatitis B and D virus strains J Clin Microbiol 2000 38 9 3311 6 10970376 10.1128/jcm.38.9.3311-3316.2000 PMC87379

[B21] Lok AS Heathcote EJ Hoofnagle JH Management of hepatitis B: 2000--summary of a workshop Gastroenterology 2001 120 1828 1853 10.1053/gast.2001.24839 11375963

[B22] Fattovich G Bortolotti F Donato F Natural history of chronic hepatitis B: special emphasis on disease progression and prognostic factors J Hepatology 2008 48 335 352 10.1016/j.jhep.2007.11.011 18096267

[B23] Heidrich B Deterding K Tillmann HL Virological and clinical characteristics of delta hepatitis in Central Europe Journal of Viral Hepatitis 2009 16 883 894 10.1111/j.1365-2893.2009.01144.x 19566789

[B24] Seetlani NK Abbas Z Raza S Prevalence of hepatitis D in HBsAg positive patients visiting liver clinics J Pak Med Assoc 2009 59 434 437 19579728

[B25] Giovanni BG Tommaso S Maria C et al A vanishing disease? An Italian Multicentre Study Hepatology 2000 32 824 7 10.1053/jhep.2000.17711 11003629

[B26] Luby SP Qamaruddin K Shah AA The relationship between therapeutic injections and high prevalence of hepatitis C infection in Hafizabad Pakistan Epidemiol Infect 1997 119 349 56 10.1017/S0950268897007899 9440439 PMC2809008

[B27] Lindh M Horal P Dhillon AP Hepatitis B virus DNA levels, precore mutations, genotypes and histological activity in chronic hepatitis B J Viral Hepat 2000 7 258 267 10.1046/j.1365-2893.2000.00236.x 10886534

[B28] Orito E Mizokami M Sakugawa H A case-control study for clinical and molecular biological differences between hepatitis B viruses of genotypes B and C. Japan HBV Genotype Research Group Hepatology 2001 33 1 218 223 10.1053/jhep.2001.20532 11124839

[B29] Williams V Brichler S Radjef N Hepatitis delta virus proteins repress hepatitis B virus enhancers and activate the alpha/beta interferon-inducible MxA gene J Gen Virol 2009 90 2759 67 10.1099/vir.0.011239-0 19625466

[B30] Fattovich G Boscaro S Noventa F Influence of hepatitis delta virus infection on progression to cirrhosis in chronic hepatitis type B J Infect Dis 1987 155 931 935 10.1093/infdis/155.5.931 3559292

